# Network oscillations in a neural mass model induced by metabolic modulation are consistent with EEG data of neocortical epileptic seizure onset

**DOI:** 10.1186/1471-2202-14-S1-P251

**Published:** 2013-07-08

**Authors:** Florian A Dehmelt, Christian K Machens

**Affiliations:** 1 Ecole Normale Superieure, Paris, France; 2 Champalimaud Neuroscience Programme, Lisbon, Portugal

## 

In neocortical epilepsy, pathological high-frequency oscillations (pHFOs) are consistently observed in EEG recordings seconds before seizure onset [1]. While this correlation is robust, a causal relationship between pHFOs and seizure onset has not yet been established. It is known, however, that neuronal activity is contingent on sufficient metabolic supply, and links between hypometabolism and epilepsy were observed [2]. Interestingly, although the delay between pHFOs and seizure onset is comparable to the time scale of metabolic supply from the blood stream, the potential effect of pHFOs on metabolic energy homeostasis has never been quantified.

Neurons contributing to pHFOs are known to synchronize their firing, presumably through the opening of axonal gap junctions [3]. We argue that such changes, combined with higher firing rates of the individual neurons, represent a surge of energy expenditure for as long as a several seconds. This is liable to exceed locally available energy reserves. Reduced availability of energy carriers, such as ATP, limits the maximum firing rate of the affected population. Such metabolic constraints arising from pHFOs can alter the balance between excitation and inhibition in the network.

Using different types of neural mass models [4,5], we demonstrate that even a transient constraint on the maximum rate of one or both populations can lead to persistent limit cycle oscillations consistent with EEG data. By studying the bifurcations of our system with respect to the metabolic vulnerability of each population, we compute the likelihood of seizure onset following pHFOs. These transitions into an oscillatory state depend on the metabolically imposed imbalance between excitation and inhibition, as well as on time-dependent external input to the system. We therefore suggest that seizure initiation in this type of epilepsy may require the coincidence of both synchronized input and the temporary exhaustion of metabolic reserves, explaining why many HFOs occurring between seizures do not trigger an epileptic event.
